# Assessing the attentional demand: improvements to the experimental protocol and possible learning effects

**DOI:** 10.3389/fpsyg.2025.1640286

**Published:** 2025-09-10

**Authors:** Ilaria Di Pompeo, Martina Marcaccio, Simone Migliore, Giuseppe Curcio

**Affiliations:** Department of Biotechnological and Applied Clinical Sciences, University of L’Aquila, L’Aquila, Italy

**Keywords:** selective attention, divided attention, switching, executive functioning, cognitive flexibility

## Abstract

**Introduction:**

Attention control is a fundamental component of cognitive functioning and involves the ability to selectively process relevant stimuli, divide attention across tasks, and flexibly switch between attention demands. The present study describes the development and validation of the Attentional Demands Task (AD-Task), a new paradigm designed to measure selective attention, divided attention, and the cognitive costs associated with switching between these attentional processes. Based on and expanded upon the Switching Attentional Demands Task (SwAD-Task), the AD-Task introduces increased stimulus complexity, optimized temporal dynamics, and enhanced ecological validity.

**Methods:**

Forty-one healthy young adults (age: 21.0 ± 2.25; 33 F) completed the AD-Task along with established attentional paradigms (Oddball Task for selective attention, Dual-Task for divided attention). In a second experimental phase, the potential effects of practice were evaluated through an intensive training protocol involving 22 participants (age: 22.5 ± 2.89; 16 F).

**Results:**

Performance indices demonstrated strong convergent validity, with significant correlations in reaction times and accuracy measures across tasks. As expected, divided attention was associated with slower response times and reduced accuracy compared to selective attention, reflecting the increased cognitive load of processing multiple stimuli within a single modality. Analysis of switching costs revealed asymmetries, with selective attention displaying greater vulnerability to Task switching effects. No significant differences emerged between trained and untrained groups in overall task performance, aside from a modest improvement in target discrimination accuracy under switching conditions in the trained group.

**Discussion:**

These findings support the AD-Task as a sensitive, reliable, and repeatable measure of attentional control and flexibility. The AD-Task advances current methodologies by addressing previous limitations related to sensory modality interference and limited task complexity.

## Introduction

Knowledge about attention has evolved, revealing that this process is fundamental to human behavior and interaction with the environment (Petersen and Posner, 2012). A relevant aspect of modern life is the prevalence of multitasking, which significantly influences attentional processes and cognitive performance (Broccia et al., 2020; Madore and Wagner, 2019). Although multitasking is perceived as an important and desirable skill (Srna et al., 2018) and is becoming increasingly present in daily life (Malkoc and Tonietto, 2019), research shows how this ability to multitask has limitations (Madore and Wagner, 2019). Some skills that help with multiple tasks at once can improve with practice, but this often requires more cognitive resources (Madore and Wagner, 2019). Multitasking is a complex cognitive skill that involves the simultaneous performance of multiple tasks and may require considerable effort from various mental functions. Among the cognitive skills crucial for effective multitasking, recent research mentions executive functions such as working memory as well as inhibitory and attentional control (Redick et al., 2016; Diamond, 2013).

The ability to switch between performing one task to another is generally studied using task switching protocols (Monsell, 2003). *Task switching* involves executive functions such as cognitive flexibility and inhibition and requires top-down control to shift attention from one task to another. This paradigm, introduced by Jersild (1927), allows for analyzing control processes that organize and reallocate mental resources during task switching, revealing the mechanisms underlying our cognitive adaptability and operational efficiency in complex and dynamic contexts. Although the concept of “switching” has been extensively studied in the literature on serial task-switching, understood as switching between trials (e.g., Meiran et al., 2000; Kiesel et al., 2010; Koch et al., 2018), in the present study we use this term in a different sense. Specifically, we refer to slower and more prolonged changes in attentional demands that occur between different blocks of tasks. The so-called “switch costs” observed in traditional tasks last only a few seconds, whereas in our case the attentional changes involve longer periods of time.

Attentional resources are limited, so when they are divided among multiple tasks, performance tends to decline. For example, the quality of performance or speed with which a task is completed may decrease when attempting to perform various tasks simultaneously. This phenomenon is particularly evident in complex tasks requiring a high concentration level (Owsley, 2011). In this context, it is important to distinguish between two different but often overlapping cognitive conditions: high attentional demand and divided attention. The former occurs when a single task, or a task-change scenario, requires significant cognitive control and sustained concentration to maintain adequate performance. It is associated with processes of task set reconfiguration, signal interpretation, and inhibition of previously active task sets. As pointed out by Kiesel et al. (2010), these tasks generate longer reaction times and increased error rates when switching between tasks, a phenomenon termed “switching costs.” These costs do not cancel out even under conditions of predictability and preparedness, indicating that task-to-task switching engages additional and costly control mechanisms that cannot be fully anticipated.

Divided attention, on the other hand, refers to the simultaneous processing of multiple tasks or stimuli. The main challenge lies in the interference that arises in the presence of tasks with common characteristics, such as overlapping stimuli or response modes. According to Kiesel et al. (2010), such interference may be stimulus-based, when the same input is relevant to multiple tasks, or response-based, when different tasks require similar or conflicting motor outputs. This overlap increases cognitive load, leads to confusion between task rules, and typically results in decreased performance, even in the absence of task switching.

In sum, while both conditions demand cognitive control, high attentional demand is primarily driven by sequential task reconfiguration, whereas divided attention involves managing parallel interference.

Studies that manipulate attentional conditions show how secondary tasks can divert cognitive resources, thus impairing performance in primary tasks (Yilmaz and Kafalıgönül, 2024; Bruckmaier et al., 2020). Despite the strong involvement of attentional processes in multitasking skills, switching between different types of attentional demands, such as focusing on a single stimulus (selective attention) versus distributing attention among multiple stimuli (divided attention), has been largely neglected.

It was only in 2019 that Liebherr and colleagues introduced a task aimed at investigating the ability to switch between different tasks by specifically stressing the cognitive domain of attention, focusing on the interaction between two components of this process: selective attention and divided attention. Thus, the Switching Attentional Demands Task (SwAD-Task) was developed to investigate the capacity to adapt to varying attentional demands, including selective and divided attention, with a focus on task switching. The task consists of three phases: training (ten trial blocks, one for selective attention and one for divided attention, with feedback on correct or incorrect responses), single demand (four blocks of selective attention and four of divided attention, with two-minute breaks between blocks), and switching (four blocks of selective attention alternating with four of divided attention).

The task involves the presentation of a stimulus composed of a figure (e.g., a triangle) and a number (e.g., three). In the selective attention condition, participants must focus on one element, ignore the other, and respond using a predefined button. In the divided attention condition, they must respond to both elements with separate buttons.

Each block contains 26 trials, with target stimuli (between 5 and 8) randomly presented. The stimuli are displayed at the center of the screen for 250 ms, with a maximum response time of 1,800 ms. The interval between stimuli randomly varies between 500 and 2,300 ms. Performance is evaluated primarily through response times, and the entire task takes approximately 20 min (Liebherr et al., 2019).

The SwAD-Task, as designed by Liebherr et al. (2019), effectively evaluates participants’ ability to manage attentional flexibility between selective attention and divided attention. However, several limitations of the task design may reduce the robustness and scope of the cognitive processes assessed. The limited variety of target and nontarget stimuli may restrict the generalizability of results to more complex real-world situations. Moreover, the relatively long response times and interstimulus intervals (500 to 2,300 ms) could lessen task urgency and participant engagement. Finally, the brief 20-min duration may fail to capture long-term adaptability and fatigue effects, which are crucial for understanding sustained attentional control. These issues highlight the need for a revised design to enhance the task’s ecological validity and comprehensiveness.

Moreover, learning from practice is a key element in attentional tasks, as it contributes to improved cognitive performance and the ability to adapt strategies in response to varying demands (Bell et al., 2018; Liao et al., 2019; Yokoyama et al., 2015). The effects of practice consist of improvements in performance on cognitive tests resulting from repeated assessments. Bell and collaborators highlighted that the effects of practice are evident in cognitive tasks even when alternative forms of the same functions are used, suggesting that familiarity and repeated exposure can contribute to improved performance (Bell et al., 2018). This finding is consistent with the broader literature indicating that cognitive training improves attention and executive functions (Liao et al., 2019; Yokoyama et al., 2015). Training and regular practice can improve visual perception and attention levels. This is relevant because although chronic exposure to attention scenarios can impair performance, consistent practice can produce cumulative effects that enhance these cognitive faculties (Koçak et al., 2024). In the context of task switching, training in performing two attention-demanding tasks simultaneously has been found to produce greater improvements than training on a single task (Worden and Vallis, 2014), due to better integration and coordination of cognitive resources (Strobach, 2020). Even in the SwAD-Task, the effects of practice may be a crucial element that requires further investigation. Although Liebherr et al. (2019) did not conduct specific studies on learning effects, the literature suggests that continuous practice could significantly influence performance, representing a limitation for using the task in protocols that require multiple measurements (Bell et al., 2018; Liao et al., 2019; Yokoyama et al., 2015). Therefore, it is essential to further explore this dimension to rule out possible learning effects that may limit the applicability of the task in contexts requiring multiple measurements. The brief nature of the task by Liebherr et al. (2019), although it may avoid practice and learning effects, may not be totally ecological since in daily life, we are exposed to an increasing number of stimuli that demand our attention. It would, therefore, be necessary to adapt attentional tasks to a more real-world context, monitoring their exposure to practice effects or fatigue that would limit their application in experimental settings that require multiple measurements.

Cognitive performance, including attentional processes, is strongly influenced by sleep, sleepiness, and various psychophysiological states such as vigor and affect. Sleep deprivation results in reduced attention, working memory, and executive functions (Gillie et al., 2013). Sleep continuity and total sleep duration are known to modulate executive functions and the ability to switch between tasks (Wilckens et al., 2014). These cognitive processes are critical for maintaining attention and responding appropriately in various contexts, underscoring the importance of sound sleep architecture (Wilckens et al., 2014). In addition, poor sleep quality causes increased emotional lability that results in reduced attention. Gobin et al. (2015) propose that emotional states may interfere with cognitive function. It follows that the interplay between cognition, emotions, and affective states suggests that the consequences of sleep deprivation go beyond simple attentional deficits to include emotional regulation, which may further exacerbate cognitive impairments (Siddarth et al., 2021). Another important factor in attention studies is vigor. Understood as a fundamental psychophysiological state, various external and internal factors, including sleep quality, physical activity, and stress levels, influence it. It exerts a direct and significant impact on cognitive performance; high vigor is correlated with better performance in tasks requiring sustained attention and concentration, while states of low energy and reduced vigor have been associated with difficulty concentrating, increased errors, and decreased productivity, especially in multitasking situations (Shirom, 2011).

Based on the literature, this study aims to explore and improve the SwAD-Task (Switching Attentional Demands Task) paradigm developed by Liebherr et al. (2019), overcoming its limitations (e.g., small number of stimuli, unrealistic response times, too short duration) to increase its ecological validity and ability to measure attentional adaptation in dynamic and realistic contexts. In a second step, the role of learning and practice in the performance of the novel Attentional Demand Task (AD-Task) will be investigated, assessing whether and how much task repetition influences the results and compromises generalizability in longitudinal or multiple measurement studies. In both phases of the study, psychological and behavioral variables (such as sleep quality, sleepiness, vigor, and affect) will also be monitored to ensure that they do not influence performance.

## Phase 1: creation and validation of the attentional demand task (AD-Task)

### Aim

To develop and validate a revised version of the Switching Attentional Demand (SwAD) paradigm, we propose the Attentional Demands Task (AD-Task), which measures selective and divided attention and the ‘cognitive cost’ due to switching between them.

### Materials and methods

#### Participants

Forty-one participants (age: 21.0 ± 2.25; 33 F) completed the AD-Task, a selective attention task (Oddball-Task, OD), and a divided attention task (Dual-Task, DT). No participant reported a history of neurological or psychological disorders, and all indicated that they had normal or corrected vision and hearing. Running a *post hoc* power analysis (G*Power 3.1) to compute the achieved power, based on the effective sample investigated, the value 1-β resulted 0.938, reaching a sufficiently good level (Faul et al., 2007, 2009).

Before the experiment, all participants gave informed consent in writing and were told they could withdraw from the study without facing consequences. The investigation was approved by the Internal Review Board of the University of L’Aquila (#61/2021-2022) and was conducted according to the principles established in the Declaration of Helsinki.

#### Materials

##### Pittsburgh Sleep Quality Index

The Pittsburgh Sleep Quality Index (PSQI), developed by researchers at the University of Pittsburgh (Buysse et al., 1989) in its Italian version (Curcio et al., 2013), was used to assess sleep quality in the previous month. The questionnaire was completed digitally (or on paper when requested) before the experimental phase to select a sample of subjects with good sleep quality. It is a self-assessment test that the subject completes independently between 5 and 10 min. The questionnaire consists of 19 items with different subscales for assessment: subjective sleep quality, sleep latency, sleep duration, habitual sleep efficiency, sleep disturbances, use of sleep medication, and daily dysfunctions. Each item is rated on a scale of 0–3 and an overall score greater than 5 indicates a sleep disorder. The Italian version (Curcio et al., 2013) achieved significant internal consistency (Cronbach’s α of 0.83).

##### Karolinska Sleep Diary

Introduced by researchers of the Karolinska Institute (Åkerstedt et al., 1994), this instrument assesses sleep quality. Available in digital form (or on paper when requested), it must be completed by subjects within 30 min of awakening to increase the reliability and accuracy of responses. The questionnaire takes about 5 min to complete. It is a self-assessment tool that examines the previous night’s sleep. Specifically, it investigates several components that contribute to sleep quality, such as continuity, depth, awakenings, degree of sleep refreshment, final awakening, and any events that may have altered sleep quality.

##### Karolinska Sleepiness Scale

The Karolinska Sleepiness Scale (KSS; Kaida et al., 2006) measures the subjective level of sleepiness at a specific time of day based on the individual’s psychophysical state in the past 10 min. This self-report scale, sensitive to situational fluctuations, is used in studies on shift work, jetlag, driving abilities, attention, performance, and clinical settings and is applicable to both men and women. It helps to assess changes due to environmental factors, circadian rhythm, and drug effects, but is not widely used clinically because it does not measure ‘trait’ sleepiness. Completing the KSS takes about 5 min. Studies by Kaida et al. (2006) indicate high validity, showing strong correlations with EEG and behavioral variables, though test–retest reliability is challenging due to score variability. The KSS uses a 9-point scale (1 = extremely alert, 9 = extremely sleepy). Scores increase with the duration of wakefulness and correlate with the time of day (Åkerstedt and Gillberg, 1990).

##### Epworth Sleepiness Scale

The Epworth Sleepiness Scale (ESS) was used to assess daytime sleepiness (Johns, 1991). It is a tool for evaluating sleep and can be useful in diagnosing related disorders. The questionnaire presents subjects with everyday situations that most people encounter over time. For each situation, the subject is asked to rate their likelihood of falling asleep on a scale from 0 to 3. A total score from 0 to 9 is considered normal, while a score from 10 to 24 indicates dysfunctional sleep that needs to be examined more closely.

##### Global Vigor-Affect Scale

The Global Vigor-Affect Scale (GVAS), introduced by Monk (1989), is a psychometric tool developed to measure an individual’s perceived vigor and affect at a given time. Comprising eight items, the GVAS evaluates two main dimensions: *vigor*, reflecting perceived energy and vitality, with high scores indicative of elevated levels of positive activation; and *affect*, capturing current positive or negative emotions, with high scores associated with positive feelings such as joy and satisfaction. Used in various research contexts, including sports psychology, mental health, and general well-being studies, the GVAS is completed on paper before specific tasks, such as before the Revised-Swad administration, inviting participants to assess their current state using a Likert scale to gage the intensity of perceived emotions and vigor.

The inclusion of the sleep self-assessment instruments (PSQI, KSD, ESS, KSS) and the G-VAS scale was motivated by the great amount of literature highlighting how sleep, sleepiness, and momentary psychophysiological states affect cognitive performance, particularly attention, and were used to explore potential modulating effects and ensure the stability of these variables across experimental days.

##### Attentional Demand-Task

The original task (SwAD task, by Liebherr et al., 2019) was developed to measure two different attentional processes: selective attention and divided attention. Additionally, this tool allows for assessing the “cognitive cost” due to switching between the two different attentional demands. The stimuli were exclusively visual and were distinguished solely by two perceptual features: color and geometric shape. In this revised version, called the Attentional Demand Task (AD-Task), each stimulus consisted of two features: geometric shape (circle, square, triangle, and diamond) and color (red, blue, green, and yellow) to make the task usable by non-schooling populations. Stimuli (target or nontarget) were the same size (3 cm high by 3 cm wide) and appeared sequentially in the center of a black screen for 250 ms, followed by a black screen (up to 900 ms) during which the participant could respond by pressing a button. The inter-stimulus interval was randomized between 250 and 1,000 ms and featured a black screen with a white fixation point (“+”).

The task is composed of 16 blocks (8 blocks for selective attention and 8 blocks for divided attention), each consisting of 60 trials, preceded by a training phase with two blocks of 15 trials, one for each attentional demand.

As in the version developed by Liebherr et al. (2019), the task used is composed of two conditions: in the first condition, four blocks of a single attentional demand (either selective attention or divided attention) are presented, allowing for the evaluation of only one attentional component at a time. In the second condition, switching costs are assessed between blocks of different attentional demands. The four blocks of selective attention and the four blocks of divided attention alternate, allowing the cognitive costs associated with switching between different attentional demands to be measured.

**Figure 1:**
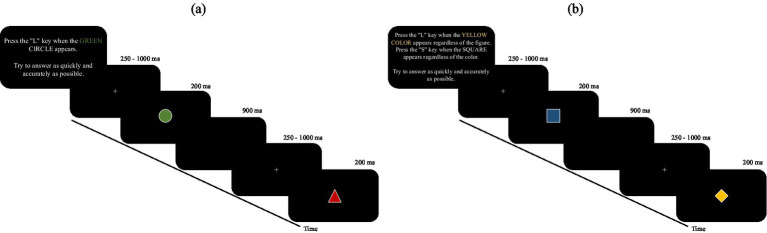
illustrates the instructions and sequence of stimulus presentation, along with the timing of task execution, for the two components of the AD-Task: **(a)** selective attention and **(b)** divided attention. The figure provides a visual overview of the procedural differences between the two conditions.

The difference between selective and divided attention tasks was explained through detailed instructions at the beginning of each block, specifying the target(s) to search for and the key(s) to press in response. In selective attention blocks, the participant must respond only when a single specific target stimulus (e.g., a green circle) appears, ignoring all other stimuli (non-target). The target stimulus is the same for each selective attention block. In the divided attention tasks, participants must respond to two distinct targets: one based on color (e.g., any red stimulus) and one based on shape (e.g., any triangle), regardless of the combination with the other feature. Before each block, detailed instructions are provided that define the criteria for identifying targets. In selective attention: press “L” only when the specific target stimulus (e.g., green circle) appears. In divided attention: press “L” when the target color stimulus appears, and press “S” when the target shape stimulus appears. The targets and response keys change from block to block and are explicitly communicated at the beginning of each session. See Figure 1 for more details.

*Selective attention.* The task consists of 4 blocks, for a total of 240 stimuli, including 174 no-target and 66 target stimuli. Specifically:

- In the first block, 45 nontarget stimuli are presented and 15 target stimuli.
- In the second block, 42 nontarget stimuli are presented and 18 target stimuli.
- In the third block, 42 non-target stimuli are presented and 18 target stimuli.
- In the fourth and final block, 45 nontarget stimuli are presented and 15 target stimuli.

*Divided attention.* The task consists of 4 blocks, for a total of 240 stimuli, including 174 nontarget and 66 target stimuli. Specifically:

- In the first block, 42 non-target and 18 target stimuli are presented.
- In the second block, 45 nontarget and 15 target stimuli are presented.
- In the third block, 42 nontarget and 18 target stimuli are presented.
- In the fourth block, 45 non-target and 15 target stimuli are presented.

*Switching.* The Switching condition consists of 8 blocks, alternating between selective attention and divided attention. Specifically, the same 4 blocks for selective attention and the same 4 blocks for divided attention are used, arranged in an alternating sequence (e.g., selective-divided-selective-divided, etc.).

To avoid a sequence effect among the different conditions presented in the task, a complete randomization of the conditions was done, by creating the following six different sequences: selective/divided/switching; selective/switching/divided; divided/selective/switching; divided/switching/selective; switching/selective/divided; and switching/divided/selective. These sequences were administered to participants in a randomized order. The total duration of the task was 50 min, and the performance was assessed by measuring reaction times and accuracy.

##### Oddball-Task, OD

To validate the divided attention condition of the AD-Task, we used an Oddball-Task. The experimental session included two different task types, each containing 360 stimuli. In one task, circles served as the target stimuli and squares as distractors, while in the other, the roles were reversed: squares were the targets and circles were the distractors (Bocquillon et al., 2012). The order of task presentation was counterbalanced, with half of the participants completing the circle task first and the other half the square task. The stimuli were solid blue shapes, presented in random order for 900 ms each, with an interstimulus interval ranging from 800 to 1,200 ms (Sanger and Dorjee, 2016; Cai et al., 2023). The stimuli were divided into three categories: standard no-target shapes (circles with a diameter of 6.66 cm or squares with sides of 6.66 cm), distractors (the opposite shape from the standard: squares or circles of the same dimensions), and target shapes (smaller versions of the standard shapes: circles with a diameter of 4.44 cm or squares with sides of 4.44 cm). The presentation probabilities were 0.84 for standard shapes, 0.08 for distractors, and 0.08 for target shapes. Before each task, participants received verbal and written instructions to respond as quickly and accurately as possible by pressing the spacebar when the target shape appeared, either a small square or a small circle, depending on the task (Bocquillon et al., 2012).

##### Dual-Task, DT

To validate the divided attention condition of the AD-Task, we used a dual-task paradigm based on a standard Go-NoGo task (modified version of Chua et al., 2017), involving two concurrent visual stimuli. Unlike conventional dual-task paradigms that combine visual and auditory cues to engage separate sensory channels, our version used only visual stimuli, focusing on a single sensory modality. Before the main test, participants completed a brief training session to familiarize themselves with the task.

The task involved two visual elements: a white square and a white circle, both displayed on a black background. Following an initial display period of 2,750 ms (pre-target phase), the square changed color, and the circle was replaced by a number from 1 to 6. Participants were instructed to respond by pressing the C key if the square turned orange and the N key if the circle displayed an even number (2, 4, or 6). Participants were also required to inhibit responses if the square turned any color other than orange (e.g., blue, red, or green) or if the circle displayed an odd number (1, 3, or 5). As with other attention tasks, participants were encouraged to respond as quickly and accurately as possible.

The task included 50 target stimuli:

- 25 color-Go stimuli (displayed for 750 ms each),
- 25 number-Go stimuli (displayed for 750 ms each), as well as 50 No-Go stimuli (750 ms each).

The task lasted 20 min in total and the performance was assessed by measuring reaction times and accuracy.

### Procedure

The study, conducted in the Laboratory of Cognitive and Behavioral Sciences (LabSCoC) at the University of L’Aquila involved participants selected according to the PSQI scores, excluding those with poor sleep quality to avoid possible confounding effects. The selected 27 participants completed the Karolinska sleep diary (KSD) throughout the experimental week to monitor the quality and duration of their sleep.

For experimental purposes, the most suitable room was initially chosen to create an optimal experimental environment, isolated from outside distractions and interference, and participants were seated at a 35.4 × 19.9-cm computer station. The protocol took place over three consecutive days at the same time each day. Before each session, participants completed the ESS, the KSS, and the G-VAS to measure, respectively, daytime sleepiness, current sleepiness, and vigor-affect.

Participants were then randomly assigned to one of three computerized attentional tasks: the AD-Task to assess selective and divided attention and switching between them; the Oddball Paradigm to measure selective attention and response to rare stimuli; and the Dual-Task Paradigm to evaluate the efficiency of performing two simultaneous tasks.

Before starting each experimental session, the participants were instructed verbally and with written instructions about the nature of the experiment in which they participated. In addition, it was made clear to them that this was a test for evaluating attention and that they were required to maintain attention and concentration to their full potential throughout the experiment. For performance assessment, accuracy was measured using the Signal Detection Theory (SDT; Stanislaw and Todorov, 1999). The hit rate was calculated as the percentage of correctly identified targets out of the total number of target stimuli presented. False alarm rate was calculated as the percentage of commission errors (incorrect responses to nontarget stimuli). The sensitivity index d′, which reflects an individual’s ability to discriminate between signal (i.e., the target) and noise (i.e., the nontarget), was calculated as the difference between the z-transformations of the hit rate and the false alarm rate (d′ = Z(hit rate) - Z(false alarm rate)). Extreme values were corrected according to the procedure of Macmillan and Kaplan (1985). Speed was assessed based on reaction times (RTs, in ms). The protocol for each day lasted approximately 40 min.

### Data analysis

Descriptive statistics were conducted on the investigated sample for age, gender, and participants’ indices of sleep quality (PSQI, KSD).

To assess the stability of the index of sleepiness, vigor, and affect, a repeated measures ANOVA was performed in which DAY was included as a factor within the scores obtained on the ESS and GVAS questionnaires (vigor and affect). A nonparametric ANOVA for repeated measures (Friedman’s test) was performed to compare sleepiness levels among the three survey days (day 1, day 2, day 3) because KSS scores did not follow a normal distribution (Shapiro–Wilk test, *p* < 0.05 for all days).

To assess the presence of correlations between the AD-Task and the individual attentional components, as well as other measures used in the study for selective attention, including the Oddball task paradigm and the Dual-Task for divided attention, Pearson’s r was employed. A nonparametric Spearman’s correlation was conducted to examine the relationship between performance in selective (AD-Task, Odd-ball) and divided attention tasks (AD-Task, Dual Task) because the hit rate scores for selective and divided attention did not follow a normal distribution (Shapiro–Wilk test, *p* < 0.05 in all conditions).

To perform the analyses, average reaction times were calculated for each attentional demand investigated (selective/divided) in each condition considered (single demand/switching). Unlike classical task-switching studies, in this study the switching cost was calculated as the mean reaction time of all trials within the switching condition blocks that involve a change in attentional demand (i.e., from selective to divided and vice versa). Incorrect responses and non-responses were excluded from the calculation of average reaction times. Subjects with a performance below 75% were identified as outliers, hit rate and fa rate indices were considered to assess accuracy. For the single-demand condition, the mean reaction times were calculated for all four blocks. In the switching condition, however, only blocks 2 through 4 were included because the first block of each attention type is not preceded by a block of a different type and, therefore, is exempt from switching effects.

For the AD-Task task, reaction times and accuracy (d’) in the three different attentional demands were evaluated using a repeated-measures ANOVA in which condition (selective attention, divided attention, and switching) was entered as an internal factor. The repeated-measures ANOVA was used to analyze reaction time and accuracy (d’) in the single demand and switching conditions, using “attentional demand type” (selective/divided) and “condition” (single-demand/switching) as factors within. In addition, to assess how much its “costs” to switch in the two attentional demands investigated, switching costs for selective and divided attention were calculated as the difference between the demand switching and single demand conditions. The resulting switching costs were analyzed by a repeated-measures ANOVA in which “attentional demand type” was included as a within-factor. Since the accuracy index data (hit rate and fa rate) did not show a normal distribution in any of the conditions (Shapiro–Wilk test, *p* < 0.05 for all conditions), a nonparametric ANOVA for repeated measures (Friedman’s test) was used. The analysis was conducted to compare performance in the selective, divided attention, and switching tasks. Additionally, the same test was used to compare performance between single-demand and switching conditions for both selective and divided attention.

To assess the presence of potential effects from declines in vigilance, a repeated measures ANOVA was conducted in which attentional demand type (selective/divided), condition (single demand/switching), and block (1, 2, 3, 4) were entered as factors within on the accuracy (hit rate, d’, fa rate) and speed (RTs) variables. The block factor refers to the single unit of the task provided for each condition (selective attention and divided into single demand or switching) and was included to monitor any changes in performance over time.

*Post-hoc* comparisons were performed if statistical significance was reached (*p* < 0.05).

All analyses were conducted using the Jamovi program, version 2.4.14, and the level of statistical significance was set at *p* < 0.05.

### Results

#### Descriptives

The 41 participants (age: 21.0 ± 2.25; 33 F) who participated in the study reported good sleep quality in the last month as assessed by PSQI (5.45 ± 2.67, min 1 max 12), which remained stable during the week of the trial, monitored by filling out sleep diary (KSD, as highlighted in Supplementary material).

#### Stability of sleepiness, vigor, and affect indices across experimental days

The indices of sleepiness, vigor, and affect were maintained on the experimental days, as evidenced by the absence of statistically significant differences in the ESS scores (*F*_2, 80_ = 0.526, *p* = 0.593, *η*^2^*p* = 0.013), in the vigor score (*F*_2, 80_ = 0.272, *p* = 0.763, *η*^2^*p* = 0.007) and affect scores (*F*_2, 80_ = 0.0344, *p* = 0.966, *η*^2^*p* = 0.001) and in the KSS scores (*χ*^2^_2_ = 0.438, *p* = 0.804).

#### Correlations with common measures of selective and divided attention

Correlations between the revised SwAD and commonly used measures of selective attention and divided attention showed high correlations in reaction time and moderate overall performance in both single-demand and switching conditions. Table 1 presents the correlations between components of the AD-Task and similar cognitive tasks, aiming to assess the convergent validity of the instrument. Specifically, (a) shows the correlations between the selective attention component of the AD-Task and the Oddball task, while (b) reports the correlations between the divided attention component of the AD-Task and the Dual task.

**Table 1:** Correlations between the selective and divided attention components of the AD-Task and convergent measures of odd-ball task and dual task in the parameters of hit rate (a) and reaction times (b).

		(1)	(2)	(3)	(4)
(a) Correlation matrix – HIT RATE					
ADT_SELECTIVE (1)	Spearman’s rho	–			
ADT_DIVIDED (2)	Spearman’s rho	0.246	–		
ODDBALL (3)	Spearman’s rho	0.453**	0.254	–	
DUAL TASK (4)	Spearman’s rho	0.070	0.485**	0.528***	–
(b) Correlation matrix – RTs					
ADT_SELECTIVE (1)	Pearson’s r	–			
ADT_DIVIDED (2)	Pearson’s r	0.813***	–		
ODDBALL (3)	Pearson’s r	0.723***	0.585***	–	
DUAL TASK (4)	Pearson’s r	0.685***	0.696***	0.653***	–

*p < 0.05, **p < 01, ***p < 0.001.

#### Evaluation of differences between single demand blocks and switching conditions

**Figure 2:**
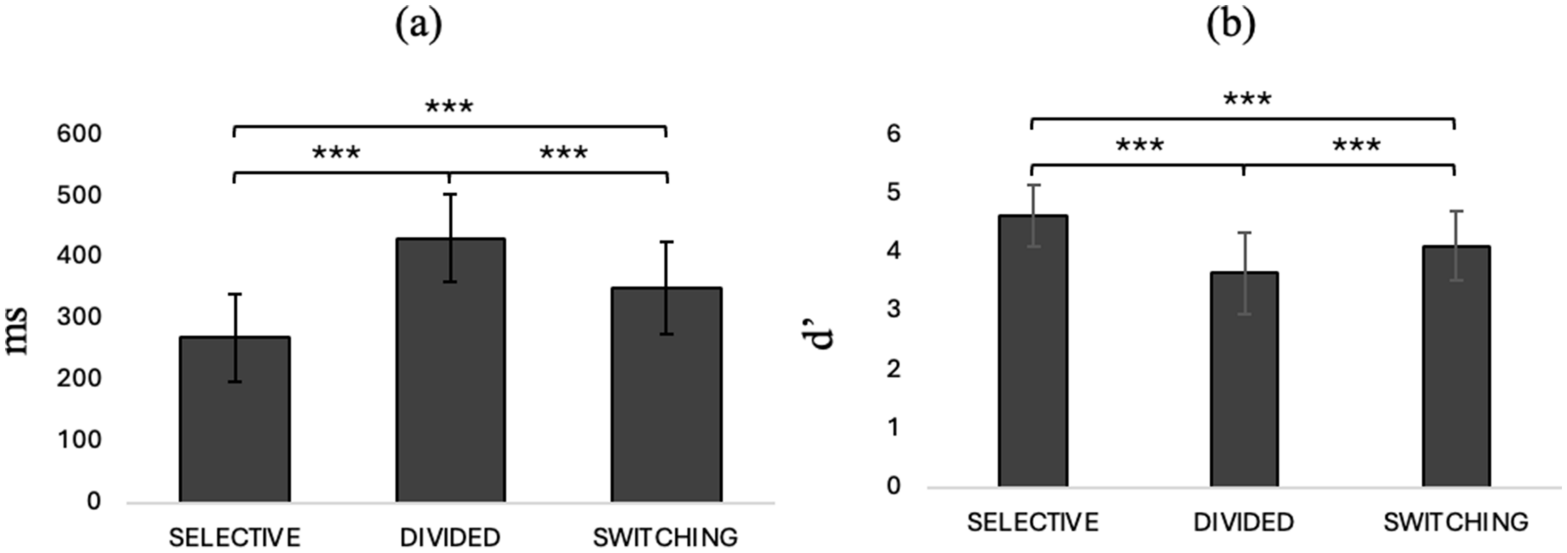
**(a)** Rts and **(b)** d’ in different conditions of AD-Task. *p < 0.05, **p < 01, ***p < 0.001.

Repeated measures ANOVA with condition (selective / divided / switching) entered as an internal factor showed a significant main effect of the condition type on speed in responding to stimuli (*F*_2, 80_ = 331, *p* < 0.001, *η*^2^*p* = 0.892) and ability to discriminate the target stimulus (*F*_2,80_ = 44.5, *p* < 0.001, *η*^2^*p* = 0.320). Post-hoc comparisons show that divided attention predicts slower reaction times and lower ability to discriminate the target stimulus than selective attention (*t*_40,0_ = 20.7, *M_dif_ =* 0.480, *p* < 0.001, *t*_40,0_ = −9.09, *M_dif_* = −0.952, *p* < 0.001) and switching attention (*t*_40,0_ = 13.3, *M_dif_* = 0.214, *p* < 0.001, *t*_40,0_ = −4.60, *M_dif_* = −0.459, *p* < 0.001). Selective attention, on the other hand, seems to be characterized by better speed and ability to discriminate the target stimulus even compared to switching (*t*_40.0_ = −16.7, *M_dif_* = −0.266, *p* < 0.001, *t*_7,0_ = 5.02, *M_dif_* = 0.494, *p* < 0.001). See Figure 2 where panel a shows RTs and panel b shows d’ in different attentional condition.

**Figure 3:**
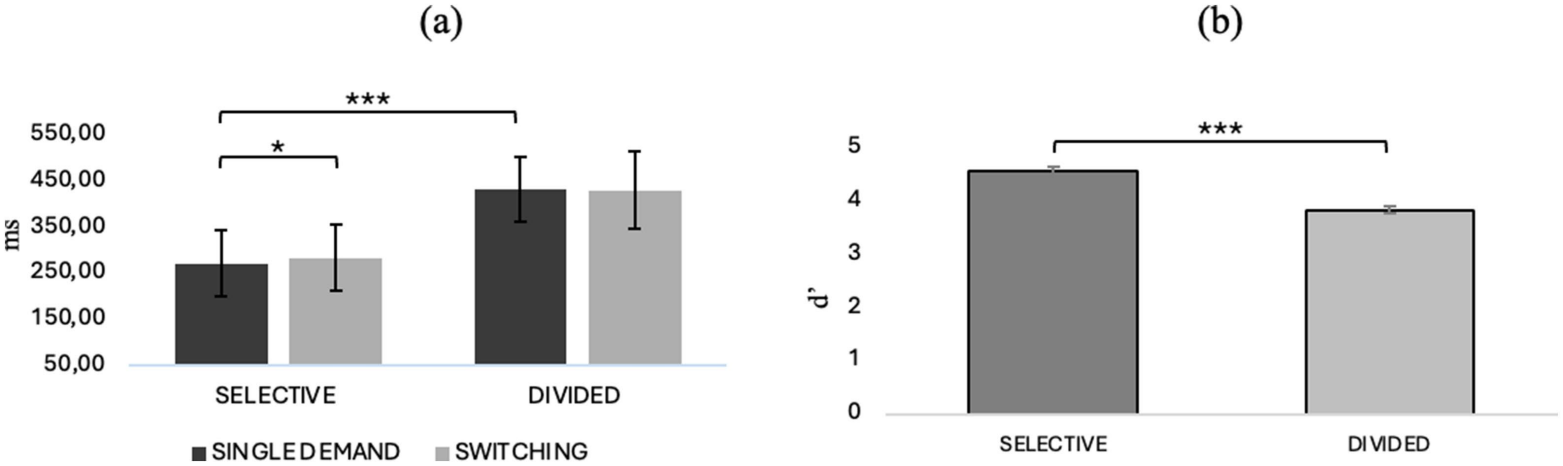
**(a)** RTs in selective and divided attention in single and switching conditions and **(b)** d’ in selective and divided attention. *p < 0.05, **p < 01, ***p < 0.001.

Repeated-measures ANOVA with “attentional demand type” (selective/divided) and “CONDITION” (single/switching demand) as factors within showed an interaction effect between attentional demand type and condition (*F*_1, 40_ = 5.87, *p* = 0.020, *η*^2^*p* = 0.128) and post-hoc comparisons show that in selective attention there is a worsening in reaction time in the switching condition compared with the single demand condition (*t*_40.0_ = −3.788, *M_dif_* = −12.44, *p* = 0.003). In addition, it also emerges that selective attention predicts better reaction times than divided attention in both the single demand (*t*_40.0_ = −21.246, *M_dif_* = −160.48, *p* < 0.001) and switching (*t*_40.0_ = −24.108, *M_dif_* = −144.34, *p* < 0.001) conditions. Repeated-measures ANOVA with “attentional demand type” (selective/divided) and “condition” (single demand/switching) as within factors on the discrimination index of target stimulus from no target showed a main effect of “attentional demand type” (*F*_1, 40_ = 103.031, *p* < 0.001, *η*^2^*p* = 0.720), showing that in general, in both the single demand and switching conditions, selective attention predicts a better ability to discriminate the target stimulus than divided attention (*t*_40, 0_ = 10.2, *Mdif* = 0.744; *p* < 0.001). For details, refer to Figure 3, which illustrates behavioral performance across various task conditions. Panel (a) displays reaction times (RTs) for selective and divided attention in both single-task and task switching conditions, while panel (b) shows sensitivity (d’) across the same conditions.

**Figure 4:**
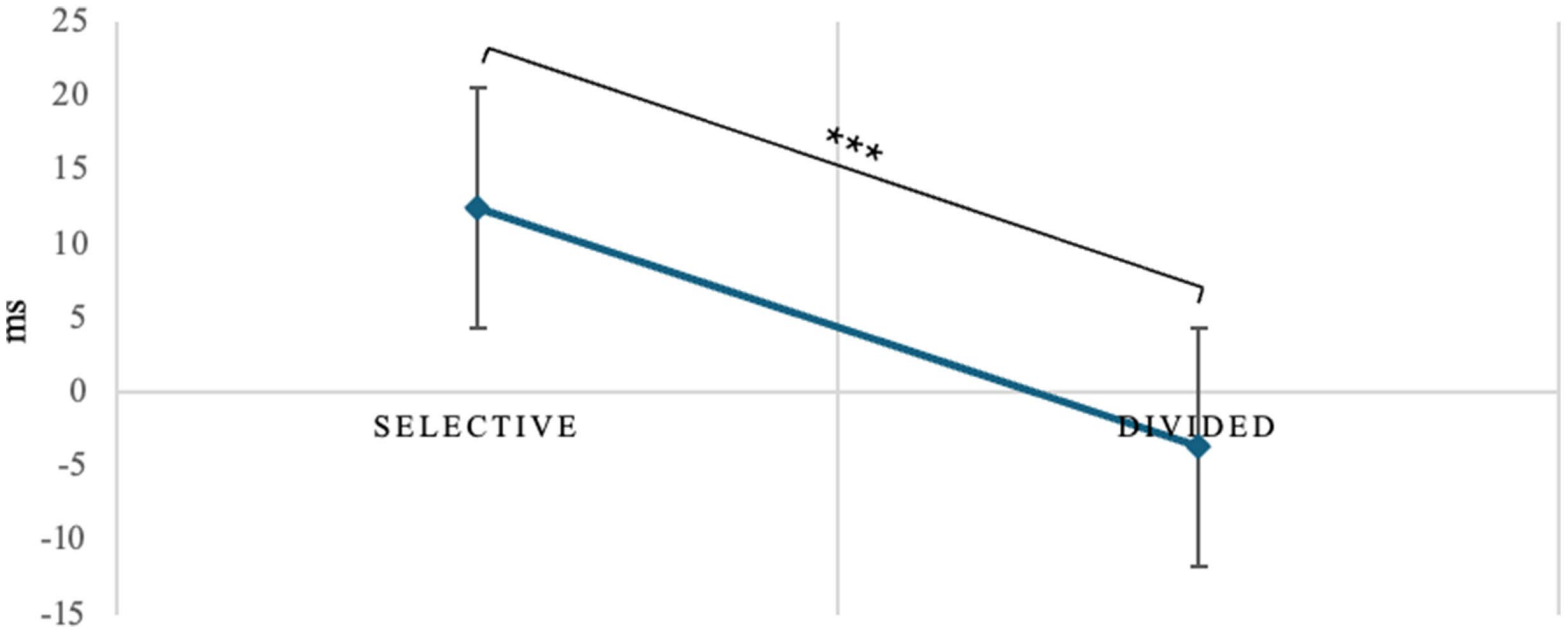
Switch cost difference between selective and divided attention. *p < 0.05, **p < 01, ***p < 0.001.

Repeated-measures ANOVA on switching costs revealed a main effect of attentional demand type (*F*_1, 40_ = 5.87, *p* = 0.020, *η*^2^*p* = 0.128), *post hoc* comparisons showing that selective attention is more sensitive to the negative effects of switching than divided attention (*t*_40.0_ = 2.42, *M_dif_* = 16.1, *p* = 0.020, Figure 4).

**Figure 5:**
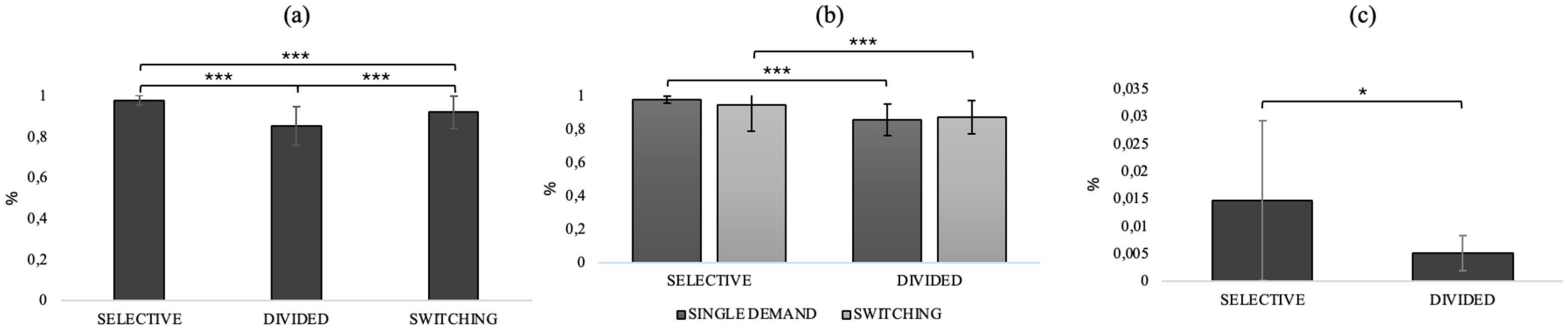
**(a)** Hit rate in different conditions of AD-Task. **(b)** Hit rate, **(c)** Fa rate on selective and divided attention in single demand and switching conditions *p < 0.05, **p < 01, ***p < 0.001.

Friedman’s test results on performance (hit rate) in the different conditions (selective/divided/switching) showed a significant effect (*χ*^2^_2_ = 62.0, *p* < 0.001), and post hoc successes show that performance in selective attention is better than both divided attention (*d* = 15.73, *p* < 0.001, selective: M = 0.98, divided: M = 0.86) and switching (*d =* 8.53, *p* < 0.001, selective: M = 0.98, switching: M = 0.93). Switching attention predicts better performance than divided (*d* = 7.20, *p* < 0.001, switching: M = 0.93, divided: M = 0.86). The results of Friedman’s test used to evaluate the differences between performance in the single demand and switching conditions for selective and divided attention showed a significant effect of attentional demand type on hit rate performance (*χ*^2^_3_ = 81.6, *p* < 0.001). Next, a post-hoc analysis was performed using the Durbin-Cover test to compare condition (single demand/switching) and attentional components (selective/divided). The results of the post-hoc analysis showed significantly better performance in selective attention than divided attention in both the single demand (*d* = 12.29, *p* < 0.001, selective attention: M = 0.98, divided attention: M = 0.86) and switching (*d* = 9.25, *p* < 0.001, selective attention: M = 0.945, divided attention: M = 0.88) conditions. No significant difference was observed between selective attention in the single demand and switching condition and between divided attention in single demand and switching. The results of Friedman’s test on the number of commission errors in the different conditions (selective/divided/switching) showed no statistically significant difference. The results of Friedman’s test used to evaluate the differences between performance in the single request and switching conditions for selective and divided attention showed a significant effect of attentional request type on the number of commission errors in the switching condition (*χ*^2^_3_ = 12.1, *p* = 0.007). Post-hoc findings using the Durbin-Cover test to compare the conditions (single/switching demand) and attentional components (selective/divided) showed a significant increase in committing errors in selective versus divided in the switching demand condition (*d* = 3.179, *p* = 0.002, selective: M = 0.014, divided: M = 0.005). No other statistically significant differences were found. Figure 5 provides an overview of performance across AD-Task conditions. Panel (a) shows the overall hit rate in different conditions of the AD-Task. Panel (b) presents the hit rate, and panel (c) the false alarm rate, for selective and divided attention under both single-task and switching conditions.

#### Assessing the presence of potential effects from declines in vigilance

The results showed no statistically significant differences in any of the variables analyzed (hit rate, reaction time, and d’), indicating stable performance throughout the task. We believe that this absence of time-related effects on task performance may be at least partly attributed to the young age of the sample, presumably associated with greater resistance to cognitive fatigue. For details, see the Supplementary material.

## Phase 2: evaluation of learning effects on the performance of the attentional demands task

### Aim

Evaluate possible learning effects from intensive task execution to consider its application in multiple measurement protocols.

### Materials and methods

In Phase 2, PSQI, KSD, KSS, ESS, G-VAS, and AD-Task were used, as described in detail in the Materials and Methods section of Study 1.

#### Participants

The learning effect was evaluated on 25 subjects, 3 of whom were excluded as outliers. The 22 participants resulting (age: 22.5 ± 2.89, 16 F) were divided into an experimental group consisting of 11 subjects (age: 21.8 ± 3.22, 8F) and a control group comprised of 11 subjects (23.2 ± 2.48, 8F). The participating subjects were recruited from students at the University of L’Aquila. All participants provided their written informed consent and were told that they could discontinue participation.

### Procedure

This study was also conducted at the LabSCoC at the University of L’Aquila. The entire protocol was performed over two consecutive days. The sample was selected based on scores obtained on the PSQI to exclude subjects with poor sleep quality. The quality and duration of sleep was monitored by filling out the KSD for four consecutive days (including the two experimental days). To obtain an appropriate experimental setting, a well-insulated classroom was selected from external noise and interference, and lighting conditions were controlled. The stations, equipped with a 35.4 × 19.9-cm computer, were spaced to isolate each subject as much as possible. The protocol required the completion of questionnaires to assess drowsiness (ESS, KSS) and vigor/affect (G-VAS) before performing the computerized task (AD-Task) on both experimental days. After completing the questionnaires (KSS, VAS, ESS), both groups performed a familiarization trial of the AD-Task on the first day of the experiment. Subsequently, unlike the control group, the experimental group underwent an intensive training session on the AD-Task, which concluded once they achieved a performance level of over 85% correct responses. On the second day of the experiment, after completing the same questionnaires (KSS, VAS, ESS), both groups performed the full version of the Attentional Demands Task. On the first experimental day, the procedure required different time commitments for each group: the control group completed the trial in approximately 5 min, while the experimental group completed the training in a variable time range of 1 to 3 h. On the second day, since both groups followed the same procedure, they completed the task in 40 min. The investigation was approved by the Internal Review Board of the University of L’Aquila (#61/2021-2022) and was conducted according to the principles established in the Declaration of Helsinki.

### Data analysis

Descriptive statistics on the sample under investigation were performed on participants’ age, sex, and sleep quality indices (PSQI, KSD), alertness, and drowsiness (GVAS, KSS, ESS). Subsequently, a *t*-test was performed to compare the two groups (1, 2) on demographic variables (age and gender). To examine possible differences between the groups in sleep quality and daytime drowsiness, a t-test was also conducted on the scores obtained in the relevant questionnaires (PSQI, ESS, KSD) administered during the sample selection phase.

The performance achieved by the two groups in the Day 2 AD-Task was compared using a T-Test for independent samples in which group (1, 2) was entered as a grouping variable on all variables investigated for both speed of execution (RTs) and performance (hit rate, d’ and fa rate). An independent sample t-test, with group (1, 2) as the grouping variable, was applied to the variables d’ and RTs for selective attention and to d’, RTs and hit rate for divided attention, under both single demand and switching conditions, to assess potential differences between the group exposed to intensive training (Group 2) and the unexposed group (Group 1).

Since the data did not meet the assumptions of normality (Shapiro–Wilk test, *p* < 0.05 in all conditions for both groups), the nonparametric Mann–Whitney test was used to compare the hit rate and fa rate scores between the experimental and control groups in the AD-Task task for divided attention, both in the single demand and switching conditions. The same test was applied to the between-group comparison for fa rate in divided attention, again in the single demand and switching conditions.

The significance level was established at *p* < 0.05. All analyses were performed using the statistical software Jamovi (version 2.4.14).

### Results

Participants in both groups reported relatively good sleep quality (group 1: M 7.55 ± 2.70; group 2: M 6.73 ± 2.53). The descriptive statistics revealed stable indices of both sleep quality and sleep restorativeness for the two groups, assessed by filling out KSD sleep diaries (as shown in Supplementary material). Descriptive statistics of the indices of sleepiness, vigor, and affect on the two experimental days are shown in the Supplementary material. The t-test for independent samples showed no statistically significant differences in the two groups in age (*t*_20.0_ = 1.112, *p* = 0.279) and scores obtained on the PSQI sleep quality assessment questionnaires (*t*_20.0_ = 0.73, *p* = 0.472), KSD and on the sleepiness and vigor indices on the two experimental days (see Supplementary material).

#### Assessment of potential learning or fatigue effects

**Figure 6:**
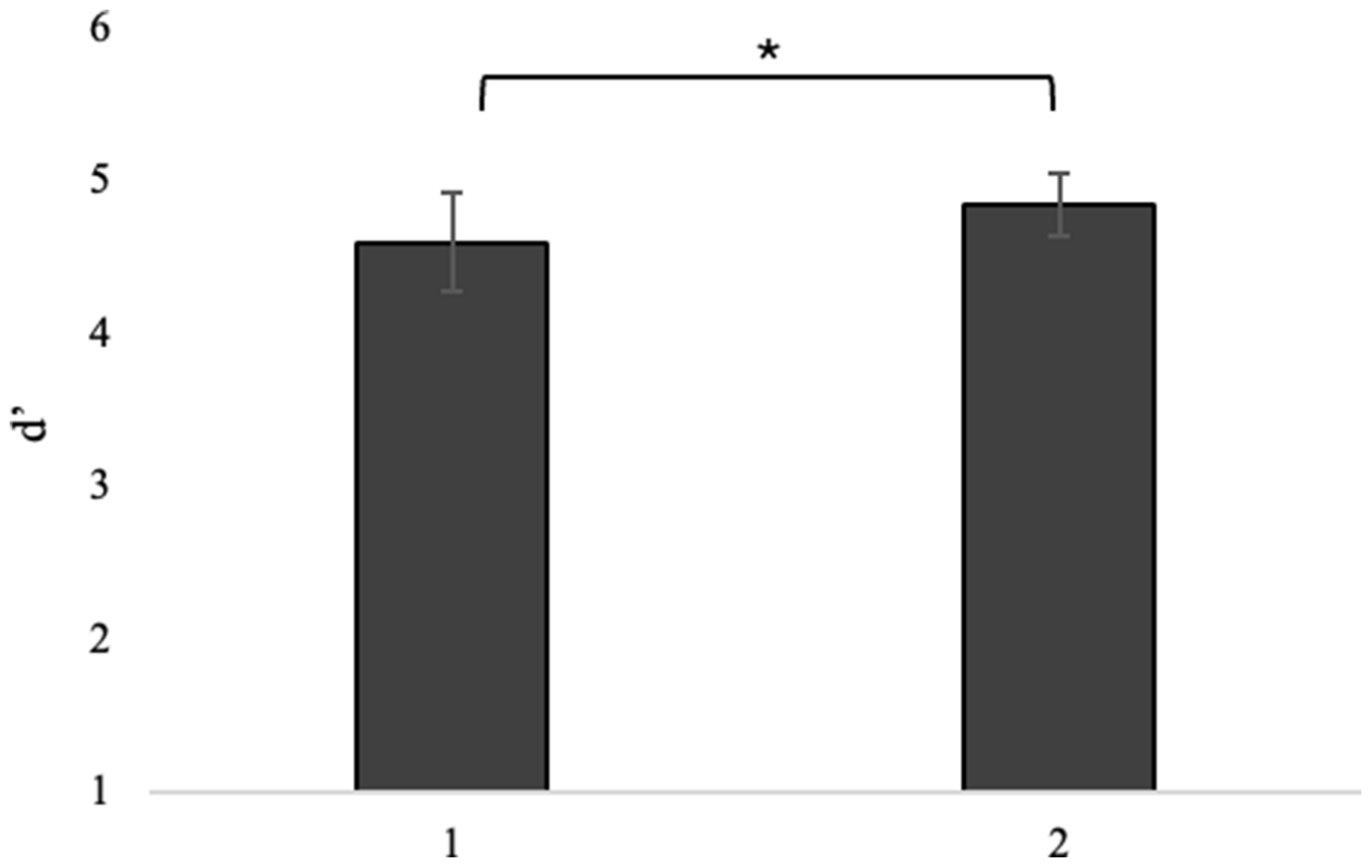
d’ in control and experimental groups. *p < 0.05, **p < 01, ***p < 0.001.

The independent-samples *t*-test used for comparison between the experimental group and the control group showed no statistically significant differences in the performance of the whole task, neither in reaction time (RT, *t*_20.0_ = −0.0850, *p* = 0.933), nor performance (hit rate, *t*_20.0_ = −1.0923, *p* = 0.288) nor discrimination indices (d’, *t*_20.0_ = −1.0492, *p* = 0.307) of the target stimulus. In selective attention, the independent samples t-test did not reveal statistically significant differences in speed, either in the single demand condition (RTs: *t*_20.0_ = −0.220, *p* = 0.828) or in the switching condition (RTs: *t*_20.0_ = −0.799, *p* = 0.434). However, a statistically significant difference was found between the training-exposed group and the unexposed group in the discrimination index for the target stimulus in the switching condition (d’: *t*_20.0_ = −2.143, *p* = 0.045, as shown in Figure 6), but not in the single demand condition (d’: *t*_20.0_ = −1.136, *p* = 0.269). The Mann–Whitney test performed on performance in selective attention found no statistically significant differences between the two groups in either the success rate in the single demand condition (hit rate: Mann- Whitney’s *U* = 56.5, *p* = 0.753) and in the switching condition (hit rate: *U* of Mann–Whitney = 47.5, *p* = 0.350) or the failure rate in the single demand condition (fa rate: *U* of Mann–Whitney = 50.5, *p* = 0.513) or in the switching condition (fa rate: *U* of Mann–Whitney = 37.0, *p* = 0.118).

Similarly, no statistically significant differences were found in divided attention when examined in the single demand condition (RTs: *t*_20.0_ = 0.456*, p* = 0.653; hit rate: *t*_20.0_ = −1.059, *p* = 0.302; d’: *t*_20.0_ = −0.320, *p* = 0.752) and in the switching condition (RTs: *t*_20.0_ = 0.427, *p =* 0.674; hit rate: *t*_20.0_ = −0.412, *p* = 0.685; d’: *t*_20.0_ = −0.181, *p* = 0.858). The Mann–Whitney test found no statistically significant differences between the two groups in the number of committing errors either in the single demand condition (fa rate: *U* of Mann–Whitney = 58.0, *p* = 0.878) or in the switching condition (fa rate: *U* of Mann- Whitney = 51.0, *p* = 0.498).

## Discussion

This study aimed to design the AD-Task, inspired by the original Switching Attentional Demands Task (SwAD-Task) for the study of selective and divided attention, which also offered the possibility of evaluating the switching costs resulting from the alternation of the two attentional components under investigation. To this end, an instrument capable of overcoming the limitations of the SwAD-Task, called the Attentional Demands Task, was developed and validated. In the same study, the influence of intensive exposure to the task was also evaluated to consider the possible existence of learning effects that could limit the application of the tool in contexts in which several exposures to the task are required. The task was tested on 41 young, healthy individuals and compared with two widely used measures to assess the same constructs. The learning effects, on the other hand, were studied in a sample of 24 subjects divided into two groups, in which the experimental group, in contrast to the control group, was subjected to intensive AD-Task training.

The results of our work can be discussed in four main sections:

(1) Correlations between AD-Task and measures commonly used to assess selective and divided attention; (2) Differences between selective and divided attention; (3) Assessment of switching costs in the two attentional components; (4) The presence of learning or practice affects the performance of the AD-Task.

Regarding the results of the comparison between AD-Task and commonly used measures of selective and divided attention, significant correlations were found in terms of speed and accuracy. These results can be considered reliable since, in the case of selective attention, an Oddball task, widely considered an effective paradigm for measuring this type of attention, was used as a concurrent measure. It involves the presentation of frequent (standard) stimuli interspersed with infrequent (target) stimuli for participants to detect and is often associated with studies evaluating the event-related potential (ERP) P300 component, which corroborates its reliability (Warren et al., 2020; Ogawa et al., 2022). Indeed, the P300 occurs approximately 300 ms after the stimulus and is a key indicator of attentional allocation and cognitive processing (Gray et al., 2004; Demirayak et al., 2023; Pavarini et al., 2018). The amplitude of the P300 is greater for rare (target) stimuli, reflecting greater attention to meaningful stimuli (Verleger and Śmigasiewicz, 2016; Ciria et al., 2017). This parameter is influenced by stimulus rarity and cognitive load (Alexander et al., 2005). The methodological limitation highlighted in Liebherr et al.’s (2019) work, which used a dual-task paradigm with stimuli belonging to different sensory domains (e.g., visual and auditory) as a competing measure for divided attention, was overcome. Unlike their Switching Attentional Demand Task, which involved only visual stimuli, the dual task involved stimuli from different sensory modalities. Switching trial-wise between different stimulus types could create interference and make it more difficult to interpret the results (Hsieh and Yu, 2003; Monsell, 2003; Hsieh and Liu, 2005). To address this critical issue, a dual-task paradigm with only visual stimuli, such as the attentional demands task, was adopted. This approach made it possible to avoid variability due to the transfer of cognitive resources between different sensory domains, ensuring greater consistency in cognitive demands and more accurate assessment in the measurement of divided attention, without introducing confounding effects related to the change of sensory domain (Hsieh and Yu, 2003; Monsell, 2003; Hsieh and Liu, 2005).

To understand cognitive performance in the two attentional components, it is important to emphasize the distinction between selective and divided attention. Selective attention consists of the ability to focus on a specific stimulus while ignoring others, whereas divided attention requires the simultaneous processing of multiple stimuli (Johnston and Dark, 1986). The results of our work showed that divided attention involves slower reaction times and worse performance than selective attention. When attention is divided, as in dual tasks, participants show reduced accuracy and slower response times (Desmarais et al., 2023; Azubike et al., 2024). One explanation for this phenomenon could be related to the use of visual stimuli alone, which may saturate available cognitive resources (Sutton et al., 1965; Wickens et al., 1983). As pointed out by Talsma et al. (2006), the processing of stimuli in the same modality is driven by reduced attentional capacity compared to processing in different modalities. Such assumptions would explain why motor and cognitive modalities beneficial effects on cognitive performance in some tasks have where motor and cognitive modalities alternate (Liebherr et al., 2018). From the literature, we know that the integration of simple motor demands into cognitive tasks, known as cognitive-motor interference (CMI), can improve cognitive performance (Al-Yahya et al., 2011). This effect is particularly observed when the motor skill is well automated, as it requires less attentional load and thus reduces interference with the cognitive task, promoting improvement in both domains (Schaefer and Lindenberger, 2013). In the case of two stimuli of the same modality, on the other hand, several empirical evidence support the idea that dividing attention between stimuli incurs perceptual costs, Niebergall et al. (2010) demonstrated that dividing attention between visual targets reduces perception compared to when focused on a single stimulus, reinforcing the idea that attentional resources are limited (Niebergall et al., 2010). Pestilli et al. (2011) also assessed that cortical responses are enhanced in early visual areas if attention is focused on a single target stimulus, compared to when it is divided over multiple stimuli. This leads to and explains reduced processing efficiency and increased cognitive load in divided attention tasks compared to selective attention tasks. Cognitive load theory suggests that increased cognitive demands, such as those experienced during task switching, can overload working memory and lead to additional cognitive costs (Lavie, 2006; Schrepel et al., 2021). Indeed, unlike attentional load, which concerns the amount of resources required to select and process relevant stimuli in the presence of distractors, working memory load involves the maintenance and manipulation of temporary information (D’Aurizio et al., 2023; Lavie, 2006).

Additional explanations regarding the differences in reaction times between selective and divided attention come from neuroimaging studies, which have shown that these two attentional components activate distinct but overlapping neural networks that would explain the variations in reaction times (Liebherr et al., 2019; Moisala et al., 2015; Pauw et al., 2019). Dual-task conditions can lead to increases in activation in brain regions already involved in single tasks, indicating that divided attention does not recruit new areas, but intensifies demands on existing networks (Moisala et al., 2015). Gharahi et al. (2023) assessed the presence of a positive correlation in reaction times between selective and divided attention tasks, where increasing demands on divided attention also predicted an increase in reaction times, reflecting the cognitive load imposed by handling multiple streams of information. The explanation may be that the right temporal hemisphere shows increased activation during divided attention tasks, underlining the implication of distinct neural pathways activated when attention is divided versus when it is selectively focused (Tomita et al., 2017). Other evidence confirming what has been argued in this section comes from studies by Weerda et al. (2006) who find greater activity in areas involved in top-down control (lateral prefrontal cortex and posterior parietal cortex, Buschman and Miller, 2007) during divided attention tasks compared to selective attention tasks, which would confirm the hypothesis of greater top-down control contributing to a prolongation of reaction time.

Finally, from the motor response point of view, as Liebherr et al. (2019) suggested, the different number of response buttons (one in the selective attention task and two in the divided attention task) could affect reaction times. This aspect, related to motor actions, might require more attention when more complex responses are needed. In selective attention tasks, where only one-key response is required, individuals focus on a single stimulus, resulting in faster reaction times. Lola et al. (2021) confirm that fewer motor demands (a single key) increase the speed of cognitive processing. In contrast, in divided attention tasks, where there are multiple response keys, the user must handle several stimuli simultaneously, increasing cognitive load and slowing the response. Liebherr et al. (2019) showed that divided attention results in longer reaction times, while Gharahi et al. (2023) point out that more response options worsen performance due to increased complexity. Studies such as those by Torre et al. (2012) and Gutiérrez-Dávila et al. (2017) also confirm that increasing the number of stimuli and response keys requires more attention management, slowing down time. Furthermore, Töllner et al. (2011) and Wilschut et al. (2011) suggest that the multiplication of response options already affects the early stages of cognitive processing.

Regarding the effects of switching between attentional demands, our results show that selective attention, which involves a lower cognitive load than divided attention (Madden and Plude, 1993), is more sensitive to switching costs. Increased attentional demand may contribute to slower response times to target stimuli in selective attention tasks, particularly when switching between selective and divided attention is involved; however, further evidence is needed to clarify this relationship (Liebherr et al., 2019). Although not statistically significant, the latter tends to show improved performance when switching to the simpler attentional component, namely the selective one. Some work shows that engaging in simpler attentional tasks before more complex ones can improve execution speed, reduce cognitive overload, improve attentional capacity and facilitate better cognitive planning (Richter and Yeung, 2015). One explanation could be related to attentional filters that adapt to better allocate resources, allowing a smooth transition to complex tasks after performing simpler ones (Wearden et al., 2010). Furthermore, mindfulness meditation, considered a simple attentional task, has been proposed in some studies as a strategy to improve attention under stress and reduce cognitive load in complex tasks (Jankowski and Holas, 2020). In support of these considerations, Nonnekes et al. (2020) highlight the importance of preparing and allocating attentional resources for performance in dual tasks.

Another explanation for our results can be traced back to inhibition theory. In some previous studies, Monsell et al. (2003) have shown that in divided attention tasks the need for inhibitory control-that is, the cognitive process that suppresses irrelevant information or responses-is reduced compared to selective attention tasks. This reduction in the need for inhibition may lead to less effective inhibitory control when returning to selective attention tasks. Furthermore, the level of inhibition may depend on how much conflict there is between possible responses: when there are more options to choose from, as in divided attention, less inhibition may be needed (Goschke, 2000). Based on these considerations, it is important to point out that proactive interference, resulting from persistent activation of the previous task and suppression of the current task, maybe a key factor in switching costs (e.g., Allport et al., 1994; Yeung et al., 2006). Indeed, the capacity-sharing model supports this relationship, arguing that when two tasks share similar attentional resources, performance on both can suffer (Patel and Bhatt, 2015). The limited capacity model suggests that attention is a limited resource and that competing tasks may impair performance in one or both tasks (Yang et al., 2018; Swerdloff and Hargrove, 2023). However, in our results switching costs are not symmetrical, switching between selective and divided attention generates different effects for each modality with selective attention being more sensitive to switching costs despite being the least complex attentional demand. This phenomenon could be related to the concept of “asymmetries in switching costs” described by Allport et al. (1994), according to which, when tasks of different difficulty are alternated, the switching cost is higher for the easier task. However, it is important to note that Allport’s results were based on trial-by-trial switching, whereas the present study involves block switching. The sequential effects of difficulty probably cause a reduction in performance after a difficult task, regardless of whether one switches to another task or repeats the same one (Schneider and Anderson, 2010). Another supporting data also comes from an increase in commissioning errors in selective attention compared to divided attention. This phenomenon could again reflect an impairment of inhibitory control, more evident in selective attention (Van Moorselaar and Slagter, 2020). Möschl et al. (2020) highlighted that under high cognitive control demands—typical of selective attention tasks—participants tend to commit a greater number of commission errors. This increase in errors suggests that the cognitive load associated with selective attention can impair its effectiveness, making it more prone to mistakes. In general, it can be inferred that the need to shift attention involves a reallocation of cognitive resources that affects selective attention more markedly, slowing its speed of response (Liebherr et al., 2019; Jefferies et al., 2021; Yilmaz and Kafalıgönül, 2024). This effect is particularly evident in contexts where a change in attentional focus is required, with a significant impact on the efficiency of selective versus divided attention (Rahman et al., 2021; Desmarais et al., 2023).

The lack of other switching effects may be due to the similarity of the two types of tasks. Studies show that the specificities of task switching costs may depend significantly on various factors, including task similarity and individual cognitive control abilities. For example, switch costs are affected by dissimilarity between task rules; greater dissimilarity typically leads to higher switch costs because more cognitive resources are required for reconfiguration during the switching process (Bustos et al., 2024). This suggests an inherent cost structure that highlights the cognitive effort involved in managing multitasking environments.

An important observation is that even in samples of young adults, performing an attentional task can lead to decrements in vigilance related to the prolonged involvement of executive control, consistent with predictions from resource control theory (Luna et al., 2022). Variables such as task duration, cognitive load, and level of difficulty can lead to decrements in vigilance, as e, as evidenced by slowed reaction time and increased errors (Luna et al., 2022). However, in our study, the absence of a decrease in vigilance could be explained not only by the young age of the sample, but also by the nature and level of difficulty of the task, as well as the possible effectiveness of the attentional strategies adopted by the participants.

Finally, the lack of significant differences in reaction time and performance between the group exposed to intensive training of the AD-Task and the unexposed group allowed us to exclude the presence of learning or fatigue effects. The only significant difference was found in the stimulus discrimination index (d’) in the switching condition for the group subjected to intensive training. This could suggest a possible sensitivity to discriminative skill practice effects. From the literature, we know that repeated exposure to cognitive tasks can improve discriminative ability (Vékony et al., 2018; Marsevani, 2022; Russo et al., 2017) but, in this case, they may be neglected as they are so marginally related to only one performance parameter and condition.

## Conclusion

In this work, the Attentional Demands Task (AD-Task), a new instrument for assessing selective and divided attention and associated switching costs, was developed and successfully validated. The AD-Task showed significant correlations with established measures, such as the Oddball task for selective attention and the Dual-Task for divided attention, confirming its reliability in analyzing the attentional constructs for which it was designed. The exclusive use of visual stimuli helped avoid confounds associated with switching between sensory modalities—common in previous task-switching paradigms—thus promoting a more consistent distribution of cognitive demands across conditions.

The results show significant differences between selective and divided attention, with the latter being associated with slower reaction times and reduced performance. This effect may reflect the involvement of greater attentional capacity required to process task-relevant stimuli under specific conditions (Lavie, 2006). This load cannot be attributed solely to the use of a single sensory modality (i.e., intramodal processing within the visual domain); in fact, even tasks that involve different sensory modalities (i.e., intermodal tasks) may require substantial cognitive resources, as evidenced by the presence of mixing costs (Schils et al., 2024).

Neuroimaging studies and theoretical models support these findings, indicating greater involvement of top-down control networks and limited attentional resources during divided attention tasks.

Regarding switching costs, an asymmetry emerged in which selective attention showed greater sensitivity in experiencing the negative effects. This asymmetry has been explained by the concept of “switching cost asymmetry” in which switching from complex tasks to simpler tasks negatively affects the simpler task (Allport et al., 1994; Schneider and Anderson, 2010). Furthermore, the absence of significant learning or fatigue effects, except for marginal improvements in stimulus discrimination in the experimental group, suggests the robustness of the AD-Task for repeated use without compromising validity.

## Limitations

Despite promising results, this study has some limitations that reduce its generalizability and applicability. First, the sample size is relatively small, which may limit statistical robustness. In addition, despite important evidence in the literature, the neural and cognitive mechanisms underlying switching costs have not been directly investigated. Future research could further investigate through neuroimaging techniques. Another limitation to mention in this study concerns some factors that could have influenced the magnitude of the observed correlations. All tasks used the same sensory modality and response format. This is especially to be considered regarding reaction time measures, which are more sensitive to general visuomotor factors than accuracy measures.

The ecological validity of the AD-Task could be improved by evaluating it in applied contexts, such as work or clinical settings, to test its usefulness in real-life situations. Finally, the modest learning effects observed suggest the need to explore further training protocols to better capture possible performance improvements.

## Data Availability

The original contributions presented in the study are included in the article/Supplementary material, further inquiries can be directed to the corresponding author.
