# Supplementary material for: Assessing the attentional demand: improvements to the experimental protocol and possible learning effects
Source: Front Psychol. 2025 Sep 10;16:1640286. doi: 10.3389/fpsyg.2025.1640286 (PMC12459298; doi:10.3389/fpsyg.2025.1640286)
Supplement: Supplementary file 1 [file Table_1.docx]

| Descriptives - Sleep Quality measured using the Karolinska Sleep Diary | | | | | | | | |
| --- | --- | --- | --- | --- | --- | --- | --- | --- |
|  | DAY 1 | DAY 2 | DAY 3 | DAY 4 | DAY 5 | DAY 6 | DAY 7 | DAY 8 |
| N | 40 | 40 | 41 | 41 | 41 | 41 | 41 | 40 |
| Missing | 1 | 1 | 0 | 0 | 0 | 0 | 0 | 1 |
| Mean | 4.45 | 4.38 | 4.30 | 4.20 | 4.23 | 4.22 | 4.10 | 4.44 |
| Median | 4.75 | 4.50 | 4.50 | 4.25 | 4.50 | 4.25 | 4.25 | 4.50 |
| SD | 0.513 | 0.499 | 0.706 | 0.736 | 0.717 | 0.628 | 0.733 | 0.539 |
| Minimum | 3.00 | 3.33 | 2.33 | 1.75 | 2.00 | 2.67 | 1.67 | 2.50 |
| Maximum | 5.00 | 5.00 | 5.00 | 5.00 | 5.00 | 5.00 | 5.00 | 5.00 |

*Table 1.1- Assessment of sleep quality evaluated by filling in the KSD sleep diary*

| Descriptives - Restorative sleep measured using the Karolinska Sleep Diary | | | | | | | | |
| --- | --- | --- | --- | --- | --- | --- | --- | --- |
|  | DAY 1 | DAY 2 | DAY 3 | DAY 4 | DAY 5 | DAY 6 | DAY 7 | DAY 8 |
| N | 40 | 40 | 41 | 41 | 41 | 41 | 41 | 39 |
| Missing | 1 | 1 | 0 | 0 | 0 | 0 | 0 | 2 |
| Mean | 3.57 | 3.67 | 3.73 | 3.11 | 3.62 | 3.48 | 3.73 | 3.44 |
| Median | 3.33 | 3.67 | 4.00 | 3.33 | 3.33 | 3.33 | 3.67 | 3.33 |
| SD | 0.771 | 0.769 | 0.962 | 0.914 | 0.820 | 0.850 | 0.756 | 0.773 |
| Minimum | 2.00 | 2.33 | 1.00 | 1.33 | 1.33 | 2.00 | 1.67 | 1.33 |
| Maximum | 5.00 | 5.00 | 5.00 | 4.67 | 5.00 | 5.00 | 5.00 | 5.00 |

*Table 1.2 - Assessment of restorative sleep evaluated by filling in the KSD sleep diary*

Table 1.3 - Repeated-measures ANOVA results with attentional demand type (selective/divided), condition (single demand/switching) and block (1,2,3,4) on reaction times

Table 1.4 - Repeated-measures ANOVA results with attentional demand type (selective/divided), condition (single demand/switching) and block (1,2,3,4) on d’

Table 1.5 - Repeated-measures ANOVA results with attentional demand type (selective/divided), condition (single demand/switching) and block (1,2,3,4) on hit rate
