# Supplementary material for: Assessing the attentional demand: improvements to the experimental protocol and possible learning effects
Source: Front Psychol. 2025 Sep 10;16:1640286. doi: 10.3389/fpsyg.2025.1640286 (PMC12459298; doi:10.3389/fpsyg.2025.1640286)
Supplement: Supplementary file 2 [file Table_2.docx]

| **Descriptives -** *Indices of sleepiness, vigor, and affect* | | | | | | | | | | |
| --- | --- | --- | --- | --- | --- | --- | --- | --- | --- | --- |
|  | group | KSS_1 | GVAS_1_V | GVAS_1_A | ESS_1 | KSS_2 | GVAS_2_V | GVAS_2_A | ESS_2 |  |
| **N** | 1 | 11 | 11 | 11 | 11 | 11 | 11 | 11 | 11 |  |
|  | 2 | 11 | 11 | 11 | 11 | 11 | 11 | 11 | 11 |  |
| **Mean** | 1 | 3.45 | 60.8 | 62.5 | 7.64 | 3.91 | 63.7 | 61.3 | 7.55 |  |
|  | 2 | 4.27 | 61.0 | 62.5 | 8.09 | 3.73 | 60.5 | 65.9 | 8.45 |  |
| **Median** | 1 | 3 | 60.0 | 65.8 | 7 | 3 | 66.8 | 60.3 | 7 |  |
|  | 2 | 4 | 54.5 | 65.0 | 7 | 3 | 64.8 | 71.8 | 7 |  |
| **SD** | 1 | 2.16 | 15.9 | 18.5 | 3.61 | 1.70 | 21.9 | 17.1 | 3.05 |  |
|  | 2 | 1.79 | 13.0 | 16.1 | 2.98 | 1.42 | 16.7 | 15.5 | 3.64 |  |

*Indices of sleepiness, vigor, and affect in the two groups during the two experimental days in the moments before the behavioral task was performed*

| **Descriptives - SLEEP QUALITY (KSD)** | | | | | |
| --- | --- | --- | --- | --- | --- |
|  | group | KSD_1_Q | KSD_2_Q | KSD_3_Q | KSD_4_Q |
| **N** | 1 | 11 | 11 | 11 | 11 |
|  | 2 | 11 | 11 | 11 | 11 |
| **Mean** | 1 | 4.23 | 4.20 | 4.02 | 4.16 |
|  | 2 | 4.14 | 4.16 | 4.18 | 4.05 |
| **Median** | 1 | 4.50 | 4.25 | 4.00 | 4.25 |
|  | 2 | 4.50 | 4.50 | 4.50 | 4.00 |
| **Standard deviation** | 1 | 0.666 | 0.472 | 0.745 | 0.785 |
|  | 2 | 0.710 | 0.635 | 0.845 | 0.678 |

*Sleep quality in the two groups in the experimental week*

| **Descriptives - SLEEP RESTORATIVENESS (KSD)** | | | | | |
| --- | --- | --- | --- | --- | --- |
|  | group | KSD_1_R | KSD_2_R | KSD_3_R | KSD_4_R |
| **N** | 1 | 11 | 11 | 11 | 11 |
|  | 2 | 11 | 11 | 11 | 11 |
| **Mean** | 1 | 3.77 | 3.36 | 3.21 | 3.36 |
|  | 2 | 3.69 | 3.24 | 3.35 | 3.43 |
| **Median** | 1 | 3.67 | 3.33 | 3.00 | 3.33 |
|  | 2 | 4.00 | 3.33 | 3.33 | 3.33 |
| **Standard deviation** | 1 | 0.772 | 0.849 | 1.00 | 0.767 |
|  | 2 | 0.817 | 0.685 | 0.754 | 0.544 |

*Sleep restorativeness in the two groups in the experimental week*

| **Independent Samples T-Test** | | | | |
| --- | --- | --- | --- | --- |
|  |  | Statistic | df | p |
| **KSD_1_Q** | Student's t | 0.310 | 20.0 | 0.760 |
| **KSD_2_Q** | Student's t | 0.191 | 20.0 | 0.851 |
| **KSD_3_Q** | Student's t | -0.468 | 20.0 | 0.645 |
| **KSD_4_Q** | Student's t | 0.363 | 20.0 | 0.720 |
| **KSD_1_R** | Student's t | 0.224 | 20.0 | 0.825 |
| **KSD_2_R** | Student's t | 0.369 | 20.0 | 0.716 |
| **KSD_3_R** | Student's t | -0.360 | 20.0 | 0.722 |
| **KSD_4_R** | Student's t | -0.241 | 20.0 | 0.812 |
| **Note. Hₐ μ _1_ ≠ μ _2_** | | | | |

| **Independent Samples T-Test** | | | | |
| --- | --- | --- | --- | --- |
|  |  | Statistic | df | p |
| **KSS_1** | Student's t | -0.96601 | 20.0 | 0.346 |
| **GVAS_1_V** | Student's t | -0.03667 | 20.0 | 0.971 |
| **GVAS_1_A** | Student's t | -0.00614 | 20.0 | 0.995 |
| **ESS_1** | Student's t | -0.32181 | 20.0 | 0.751 |
| **KSS_2** | Student's t | 0.27217 | 20.0 | 0.788 |
| **GVAS_2_V** | Student's t | 0.38332 | 20.0 | 0.706 |
| **GVAS_2_A** | Student's t | -0.66926 | 20.0 | 0.511 |
| **ESS_2** | Student's t | -0.63500 | 20.0 | 0.533 |
| **Note. Hₐ μ _1_ ≠ μ _2_** | | | | |

*Differences between the two groups in indices of sleep quality, sleepiness, vigor and affect*
